# Vitamin D and VDR gene polymorphism (*FokI*) in epithelial ovarian cancer in Indian population

**DOI:** 10.1186/1757-2215-6-37

**Published:** 2013-05-26

**Authors:** Sudhesna Mohapatra, Alpana Saxena, Gauri Gandhi, Bidhan Chandra Koner, Prakash Chandra Ray

**Affiliations:** 1 Department of Biochemistry, Maulana Azad Medical College, Bahadur Shah Zafar Marg, New Delhi, Delhi 110002, India; 2 Department of Obstetrics and Gynecology, Maulana Azad Medical College, Bahadur Shah Zafar Marg, New Delhi, Delhi 110002, India

## Abstract

**Introduction:**

Vitamin D deficiency and vitamin D receptor (VDR) gene polymorphism, *FokI*, is reported to increase the risk of many cancers. Role of vitamin D and its receptor polymorphisms in ovarian cancer has not been clearly defined.

**Objective:**

To study the levels of serum vitamin D and occurrence of vitamin D receptor gene polymorphism (*FokI*) in cases of ovarian cancer.

**Material and methods:**

*FokI* genotyping was done by PCR-RFLP technique and vitamin D levels were estimated by chemiluminescence immunoassay.

**Results:**

Serum vitamin D levels were significantly (p < 0.03) lower in ovarian cancer cases as compared to controls. The homozygous (TT) and heterozygous (CT) genotype predispose to the development of ovarian cancer in Indian population (OR: 2.37, 95% CI: 1.04-5.44) as compared to the homozygous (CC) genotype. Vitamin D deficiency and VDR gene polymorphism (*FokI*) act non-synergistically (p value < 0.4).

**Conclusion:**

Low blood levels of vitamin D and VDR receptor polymorphism (*FokI*) might be a risk factor for the development of ovarian cancer. Other novel ligands of vitamin D receptor might be responsible for the non-synergistic effect.

## Introduction

Ovarian cancer is the 6th most common cancer in women with estimated lifetime risk of 1 in 70 women [1]. Epithelial ovarian cancer is the most common histological type of ovarian cancer. More than seventy percent of these patients present in the advanced stage of this disease, and have a cure rate of less than forty percent [2]. The high mortality in these cases is due to lack of highly sensitive and specific screening methods.

Vitamin D is a fat soluble secosteroid which is involved in a wide variety of biological processes like bone metabolism, modulation of immune response, cell proliferation and cell differentiation. There exists an inverse relationship between vitamin D levels in blood and incidence of many cancers [3,4]. The studies conducted by Arslan *et al*. and Tworoger *et al.* couldn’t establish any direct relationship between vitamin D deficiency and risk of ovarian cancer [5,6]. But Tworoger *et al.* reported that vitamin D deficiency is associated with significant risk of ovarian cancer in overweight and obese women [6]. Activity of vitamin D is mediated by vitamin D receptor (VDR). VDR gene polymorphism, *FokI*, (rs10735810/rs2228570) is reported to be in linkage disequilibrium with other VDR polymorphisms. A change in the sequence from C to T in the start codon translation site leads to generation of a polymorphic variant (TT) which is three amino acids longer and has decreased transactivation capacity as compared to the short CC allele [7]. Several population based studies indicated that VDR gene polymorphisms are associated with human cancers [8,9]. A few studies tried to establish a relationship between vitamin D receptor gene polymorphism (*FokI*) and ovarian cancer. The odds ratio in these studies were observed to vary from 1.09 to 2.5 indicating that CT and TT genotypes of VDR gene polymorphism (*FokI*) are at increased risk of ovarian cancer[10-13]. However there is hardly any data in this regard in the Indian population. Hence the present study was designed (a) to evaluate the levels of serum vitamin D in epithelial ovarian cancer patients, (b) to evaluate the association of Vitamin D receptor (VDR) gene polymorphism (*FokI*) with the risk of epithelial ovarian cancer and (c) to explore if the relationship between vitamin D levels and vitamin D receptor polymorphism *FokI* is additive in their action.

## Material and methods

A case control study was designed to recruit fifty subjects in each group and conducted in the department of Biochemistry and department of Obstetrics and Gynecology, Maulana Azad Medical College, New Delhi. Written informed consent was taken from the cases and controls. Blood sample (5 ml) was collected from fifty newly diagnosed ovarian cancer patients who had histopathologically confirmed epithelial ovarian cancer. The study group was subjected to a structured questionnaire (regarding demographic, medical, lifestyle and reproductive information). Fifty controls were matched with respect to age, menopausal status and month of blood draw. The study was approved by the institutional ethics committee of Maulana Azad Medical College, New Delhi.

### Serum vitamin D estimation

The serum vitamin D was measured by electrochemiluminescence immunoassay method using Elecsys Total Vitamin D (25-OH) kit (Roche diagnostics, Mannheim, Germany) adapted to ELECSYS 2010 (Roche diagnostics, Mannheim, Germany).

### Genotype analysis

**Figure 1:**
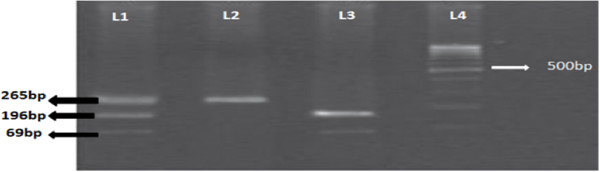
***Fok I *Restriction digested PCR product of VDR gene. L4-100 bp molecular weight marker; L3-Homozygous TT genotype, L2-Homogygous CC genotype, L1-heterozygous CT genotype.**

Genotyping was performed without the knowledge of the case/control status of the study subjects. Genomic DNA was extracted from blood samples collected in EDTA vials using DNA sure blood mini kit (Nucleo-pore, Genetix Biotech Asia Pvt. Ltd., New Delhi, India) according to the manufacturer’s instructions. VDR *FokI* genotype was analyzed using PCR- RFLP. The DNA was amplified by polymerase chain reaction using primers described by Harris *et al.*[14]. The primers used for PCR–RFLP were Forward 5′- AGCTGGCCCTGGCACTGACTCTGCTCT -3′ and Reverse 5′- ATGGAAACACCTTGCTTCTTCTCCCTC -3′ resulting in a PCR product of 265 bp. The amplification was accomplished with a 50 μl reaction mixture containing 5 μl of 20 ng template DNA, 0.25 μl 25 pmol of each primer, 2.5 μl 10 mM dNTPs, 1.5 μl of 20 mM MgCl_2_, 0.3 μl of 5 U/ μl Taq polymerase with 2.5 μl of 10X Taq Buffer (Fermentas, MA, USA). PCR conditions were as follows: Initial denaturation at 94°C for 10 minutes followed by 35 cycles of denaturation at 94°C for 45 seconds, annealing at 60°C for 45 seconds, extension at 72°C for 45 seconds and final extension at 72°C for 5 minutes. The amplicons were digested with 4 units of *FokI* enzyme (NEB, MA, USA) by incubating at 37°C for 4 hours. The presence of T allele created a restriction site in the amplified region which was cut by *FokI* to produce 2 fragments of 69 bp and 196 bp visualized on 3.5% agarose gel containing ethidium bromide (Figure 1).

### Statistical analysis

Statistical analysis was done with SPSS version 17.0 (SPSS, Inc., Chicago IL). Independent T test (for parametric data) and Mann Whitney U test (for nonparametric data) were used to compare the data. The relationship between vitamin D and ovarian cancer was determined using logistic regression. The associations between CT and TT genotypes and risk of ovarian cancer were estimated by computing the odds ratios (ORs) and their 95% confidence intervals (CIs). Synergy factor was calculated to measure the interaction between vitamin D deficiency and VDR polymorphism *(FokI)*[15]. Statistical difference was considered significant for p values <0.05.

## Results

### General characteristics of study population

The age, parity, menopausal status, family history of relevant cancers and method of contraception among cases and healthy controls are summarized in Table 1. Ovarian cancer patients were between the age group 20–80 years with the mean age being 47 years. There were three patients in stage I ovarian cancer, four patients in stage II, forty in stage III and three patients in stage IV. Six patients had grade I (well-differentiated) tumour, twenty nine had grade II (moderately differentiated) tumour and fifteen had grade III (poorly- differentiated) tumour.

**Table 1:** Characteristics of epithelial ovarian cancer cases and controls

	**Ovarian cases (n = 50)**	**Controls (n = 50)**	p value*
Age in yr	47.9 ± 13.35	47.2 ± 12.4	
Menopausal status			
Pre-menopausal	19 (38%)	20 (40%)	
Post-menopausal	31 (62%)	30 (60%)	
Subjects with positive family history of breast or ovarian cancer	3	1	0.50
Parity:			
Nulliparous	4	1	0.18
1	4	4	0.13
2-3	29	36	0.14
≥ 4	13	9	0.23
History of use of oral contraceptives	4	6	0.37
Tubal ligation	11	5	0.08

* by chi square test.

### Serum vitamin D levels

**Figure 2:**
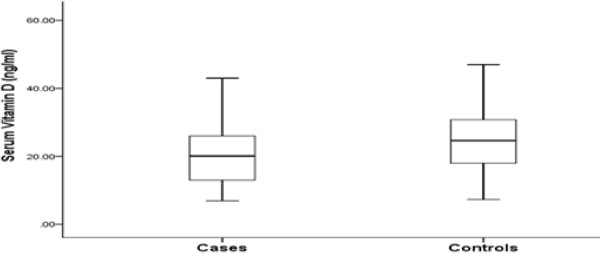
Box plot showing the distribution of vitamin D levels in ovarian cancer cases and controls.

The median of serum vitamin D levels in cases were 20.1 ng/ml which was significantly (p value <0.03) lower than that in controls (24.6 ng/ml) (Figure 2). Women with low vitamin D levels (bottom 33%) were at a higher risk for epithelial ovarian cancer (OR:3.0; CI: 1.01-7.40; p value < 0.05) than those with high levels (top 33%) (Table 2). Serum vitamin D levels in ovarian cancer patients who were in the reproductive age group were not significantly different from that of control subjects in the same age group. But the levels were significantly different between cases and controls in post-menopausal group (Table 3). There was no significant difference with respect to other parameters like clinical staging and histological grading (Table 4).

**Table 2:** Odds ratio (OR) and 95% CI for ovarian cancer according to tertile levels of baseline vitamin D levels

**Median (Range) of serum vitamin D levels in ng/ml**	**Ovarian cancer patients**	**Controls**	**OR (95% CI) Unadjusted**	p value*
Top tertile 36 (27–47)	17	22	1	
Bottom tertile 11.9 (5–16.2)	16	10	3.0 (1.01-7.40)	< 0.05

* by logistic regression.

**Table 3:** Median (range) of serum vitamin D (in ng/ml) in reproductive and post-menopausal age group in ovarian cancer patients and controls

	**Ovarian cancer patients**	**Controls**	p value*
**Reproductive age group**	18.3 (7.2-61.8)(n = 19)	21.4 (7.3-40)(n = 20)	0.41
**Post-menopausal women**	20.6 (6.93-43)(n = 30)	27.8 (7.7-47)(n = 31)	0.03

* by Mann Whitney U test.

**Table 4:** Comparison of vitamin D levels in different clinical stages and histological grading in ovarian cancer

	**Median (Range) of vitamin D in ng/ml**	p value*
**Clinical staging**		
Stage I and II (n = 7)	18.7 (12.8,30.5)	0.855
Stage III (n = 40)	20.8 (6.9,61.8)	
Stage IV (n = 3)	14 (12.9,26)	
**Histological grading**		
Well-differentiated (n = 6)	16.7 (11,24.7)	0.442
Moderately differentiated (n = 29)	20.6 (6.9,61.8)	
Poorly-differentiated (n = 15)	17 (7.2,37)	

* by Kruskal wallis test.

### Genotype distribution

The genotyping results are shown in Table 5. In the case group, 26 patients had FF genotype, 19 had Ff genotype (heterozygous) and 5 patients had the mutant ff genotype. In the control group, 36 had FF genotype, 10 had Ff genotype and 4 had ff genotype. Distribution of VDR genotype was significantly different (χ^2^ = 4.24, p value < 0.05) in ovarian cancer patients from that in controls.

**Table 5:** Distribution of FF and Ff/ff genotypes in cases and healthy controls

	**Cases**	**Healthy controls**	**Chisquare (df)**	**p value**	**Odds ratio (95% CI)**	p value*
**CC(FF)**	26 (52%)	36 (72%)	4.24(1)	**<0.05**	1	
**CT + TT (Ff + ff)**	24 (48%)	14 (28%)			2.37 (1.04-5.44)	**<0.05**

*by logistic regression.

By unconditional logistic regression analysis, it was found that in comparison to the CC genotype, the CT and TT genotype (combined) were at significantly higher risk of ovarian cancer (OR = 2.37, 95% CI 1.04-5.44, p < 0.05).

To measure the combined effect of vitamin D deficiency (vitamin D <20 ng/ml) and *FokI* polymorphism, we calculated their synergy factor which was not found to be statistically significant (Table 6).

**Table 6:** **Synergy factor (SF) in ovarian cancer between serum vitamin D and VDR (*Fok1) *gene polymorphism**

**VDR gene (*Fok1*)**	**Vitamin D deficient**	**Controls**	**Ovarian cancer cases**	**OR**	**SF (p value)**
-	-	25	15	Reference	2.1 (0.4)
+	-	10	10	1.67	
+	+	4	14	5.83	
-	+	11	11	1.66	

## Discussion

Vitamin D was known to be involved in bone metabolism but its role in other diseases like cancer, autoimmune diseases and diabetes mellitus is being studied extensively only in recent times. Ovarian cancer, on the other hand is a disease whose etiology is attributed to incessant ovulation and hormonal imbalance. Potential role of vitamin D in cancer prevention has been widely described [16-18]. There exist numerous studies which show inverse relationship of cancers of different organs with sun exposure including ovarian cancer [19-21]. However only a few studies have evaluated the role of serum vitamin D levels in ovarian cancer, and most are on Caucasian population [5,6]. To our knowledge this is the first study showing a clear relationship between vitamin D deficiency, VDR functional polymorphism (*FokI*) and risk of ovarian cancer in Indian population.

It may seem surprising to see the prevalence of vitamin D deficiency in a tropical country like India. The reason behind this could be the lifestyle of people where most of the women stay indoors. The poor intake of dairy products due to social factor and dietary habits may also contribute to this. A few studies conducted on general prevalence of vitamin D deficiency in India show alarming trends [22,23]. In our study, the participants in the highest tertile had a significant lower risk of ovarian cancer than those in the lowest tertile. On subset analysis, the mean vitamin D level of ovarian cancer (21 ± 9.1 ng/ml) was significantly lower than that of controls (26.5 ± 8.5 ng/ml) in the post-menopausal group. The reason could be that in post-menopausal state there is an increased need of vitamin D due to decreased expression of VDR caused by decrease in estrogen levels [24].

In the present study, we have observed that VDR gene polymorphism (*Fok1)* is associated with the risk of developing ovarian cancer. *FokI* (rs2228750) is a coding nonsynonymous single nucleotide polymorphism (SNP) in the translation initiation code that has been reported to have functional significance in several in vitro studies [25]. This polymorphism is considered to be an independent risk marker as it has not been reported to be in linkage disequlibrium with other VDR polymorphisms [26]. In HELA cells, transcriptional activation studies using a reporter construct under the control of a short portion of the rat 24-hydroxylase gene promoter region (−291- + 9) containing a vitamin D responsive element (VDRE) have shown that the short 424 amino acid VDR protein variant to be more active than the long 427 amino acid variant [27]. In a study with MCF-7 breast cancer cell line, it was seen that VDR-ff and VDR-FF expressing cells were morphologically similar, but the VDR-FF variant is more efficient in mediating 1,25 (OH)_2_ D_3_ action. The reason for increased vitamin D efficacy in VDR-FF was probably due to increased VDR protein stability. VDR-FF cells were resistant to the effects of the protein synthesis inhibitor cycloheximide even without 1, 25(OH)_2_ D_3_ treatment, indicating that the VDR-FF protein may be more stable than VDR-ff protein [28]. In conclusion, both protein stability and higher activity of the VDR-FF variant contribute to this variant’s enhanced response to vitamin D. Our results show that the CT and TT genotype were associated with a twofold increase in ovarian cancer risk. Other studies that have explored the relationship between VDR gene polymorphism (*FokI)* and risk for ovarian cancer are summarized in Table 7.

**Table 7:** **Summary of other studies on *Fok I *in ovarian cancer**

**Study**	**Study population**	**Patient/control participant**	**Odds ratio FFvsFf/ff**	**C.I (95%)**	**p value**
Lurie et al. 2007 [10]	U.S				
	Caucasian	71/144	**FF** 1	Reference	0.04
	Japanese	93/172	**Ff** 2.5	1.3-4.8	
			**ff** 2.1	0.8-5.2	
			**FF** 1	Reference	0.87
			**Ff** 1.2	0.7-2.0	
			**ff** 0.9	0.4-2.2	
Clendenen et al. 2008 [11]	U.S + Sweden	168/321	**FF** 1	Reference	0.55
			**Ff** 1.10	0.67-1.81	
			**ff** 1.23	0.61-2.51	
Tworoger et al. 2009 [12]	U.S	1473/2006	1.16	1.00-1.35	0.03
Lurie et al. 2010 [13]	U.S	1820/3479	1.14	1.01-1.28	0.03

The increased risk of ovarian cancer in combined vitamin D deficiency and vitamin D receptor polymorphism is expected to be due to modulation of same target molecules. But we observed that low serum vitamin D levels along with homozygous TT allele didn’t lead to synergistic increase in the risk of epithelial ovarian cancer (synergy factor:2; p value < 0.4). There are other novel ligands of vitamin D receptor and co-modulators influencing vitamin D signaling mechanism [29-31]. The non-synergistic effect indicates that these novel ligands of vitamin D receptor and co-modulators might also play a role in determining the risk of ovarian cancer which is worth exploring.

## Conclusion

It is suggested that low vitamin D levels might be a risk factor for ovarian cancer. Additionally, VDR gene (*FokI*) polymorphism may be a genetic modifier for ovarian cancer risk in Indian population. The homozygous *FokI* (TT) and heterozygous (CT) polymorphism and vitamin D levels have independent effect on cancer development and are not synergistic in their actions. However, independent large population-based prospective studies are needed to validate our findings and to facilitate rigorous analyses of subgroups. Thus our study provides evidence that the protective effect of vitamin D supplementation against ovarian cancer (especially in postmenopausal women) is worth investigating in Indian population.

## Competing interests

The authors declare that they have no competing interests.

## Authors’ contributions

SM was involved in processing of samples and molecular genetics work and drafting the manuscript. AS was involved in carrying out molecular genetics studies.GG was involved in collection of samples, clinical staging of cases and drafting the manuscript. BCK was involved in statistical analysis, drafting and correction of manuscript. PCR was involved in carrying out molecular genetics studies, ELISA and correction of manuscript. All authors read and approved the final manuscript.
